# Interactive Vision–Based 3D Augmented Reality System for In-Home Rehabilitation in Older Adults Following Total Joint Replacement: Cross-Sectional Survey of Patients and Service Providers

**DOI:** 10.2196/87572

**Published:** 2026-04-20

**Authors:** Adriana C Salvia, Siona Phadke, Manuela Kunz, Afolasade Fakolade

**Affiliations:** 1 School of Rehabilitation Therapy, Queen's University, Louise D Acton Building, 31 George Street, Kingston, ON, K7L 3N6, Canada, 1 6135336000 ext 77893; 2 Digital Technologies Research Center, National Research Council Canada, Ottawa, ON, Canada

**Keywords:** arthroplasty, older adults, exercise prescription, post-exercise recovery techniques, telerehabilitation, vision-based interactive tools, gamification, stakeholder participation

## Abstract

**Background:**

Completing home exercise programs remains a significant challenge for older adults after total joint replacement (TJR), often resulting in suboptimal rehabilitation outcomes. Augmented reality (AR) technologies offer a promising approach to addressing limitations associated with traditional paper-based home exercise programs.

**Objective:**

This study aimed to identify priorities for design features and practical or logistical considerations to inform the future design of a 3D AR system (3D ARS) to support home-based rehabilitation for older adults following TJR.

**Methods:**

A cross-sectional online survey was administered via Qualtrics. The survey included items across four domains: sociodemographic and clinical characteristics, proposed design features, practical considerations, and logistical details, and potential barriers to access and use of the 3D ARS. Descriptive statistics (frequencies and percentages) were computed, and the Mann-Whitney *U* test was used to examine differences in feature preferences between older adults and service providers.

**Results:**

A total of 145 participants (56 older adults with TJR and 89 service providers) completed the survey (response rate 88.4%). Older adults and service providers shared similar priorities for several design features of the 3D ARS, including embedding individualized exercise programs, providing customized feedback on exercise progress, and including background music during exercise (all *P*>.05). However, significant differences emerged for other features. Older adults ranked the ability to receive positive, encouraging messages during exercises (*U*=1525, *P*<.001), voice activation prompts (*U*=1145, *P*<.001), and the ability to pause and resume exercises at any time (*U*=1843.50, *P*=.01) as higher priorities than service providers. Most older adults preferred real-time visual prompts to guide joint range of motion during exercise (42/56, 75%), whereas most service providers preferred chart-based progress reports (63/89, 71%). Participants further recommended accessing the system through physiotherapy clinics, initiating use within the first month following surgery, and maintaining access for 6‐12 months.

**Conclusions:**

Findings identify key user-informed priorities for the design of AR-based rehabilitation technologies for older adults following TJR, including personalized exercise programming, accessible system interfaces, and timely feedback mechanisms. Incorporating these priorities into future system development may support scalable models of home-based rehabilitation that complement conventional physiotherapy care.

## Introduction

Total joint replacement (TJR) is a well-established surgical intervention for older adults with advanced osteoarthritis when conservative management strategies are no longer effective [1]. Advances in surgical techniques, perioperative care, and enhanced recovery pathways have shortened hospital stays, and most patients (74%‐96%) are now discharged directly home after surgery [2-4]. After discharge, patients are typically prescribed home exercise programs to restore joint range of motion, muscular strength, and functional mobility [5]. However, maintaining adherence to home exercise programs remains challenging for many older adults after TJR, often resulting in delayed functional recovery and reduced quality of life [6,7]. Commonly reported barriers include limited access to supervision and feedback once patients return home [8,9], difficulties recalling or correctly performing prescribed exercises [10], low motivation [11,12], and challenges integrating structured exercise into daily routines [9,11]. Additional contextual factors, such as variability in home environments [13,14], limited social support [15], and geographic or transportation barriers to outpatient physiotherapy services [16], may further constrain engagement in rehabilitation for some older adults after TJR. Conventional paper-based home exercise programs, while inexpensive and widely used, often provide static instructions that lack personalization, interactivity, and real-time performance feedback, which may further limit motivation and adherence [17,18].

Emerging augmented reality (AR) technologies present a promising avenue for addressing some of these challenges. AR systems overlay virtual images and information onto the real-world environment, allowing users to interact with digital content while remaining grounded in their physical surroundings [19,20]. In rehabilitation contexts, AR has been explored as a means of delivering guided exercise, enhancing motor learning through visual and auditory feedback, and supporting task-specific practice within meaningful environments [21-24]. This approach contrasts with fully immersive virtual reality (VR), which replaces the real-world environment entirely and may not be suitable or acceptable for all older adults due to issues, such as disorientation or “cybersickness” and physical discomfort (eg, neck pain) sometimes caused by VR headsets [25,26].

While many contemporary AR applications are delivered using smartphones or head-mounted displays, including optical see-through devices, these platforms may pose usability, cost, and comfort barriers for some older adults, particularly in unsupervised home settings [27,28]. In contrast, AR delivered through familiar nonwearable displays, such as tablets or televisions equipped with cameras, depth sensors, or computer-vision capabilities, may represent a more accessible and acceptable configuration for home-based rehabilitation [21,22,29,30]. In these systems, digital overlays, avatars, or visual cues are superimposed onto a live or screen-based representation of the user’s environment, preserving core characteristics of AR while avoiding the need for head-mounted equipment. Although the use of AR glasses as an alternative to the TV or monitor offers a more immersive experience, they can also trigger severe symptoms of visually induced motion sickness and dizziness [31] and might therefore not be suitable for safe use for older adults in a home environment. For older adults, leveraging familiar household devices may reduce cognitive and technical burden, enhance perceived ease of use, and support sustained engagement with rehabilitation technologies [27,32].

One configuration that may support accessible home deployment involves plug-and-play camera-based AR systems that connect directly to household televisions or monitors. Similar systems are already widely used in motion-based gaming. For example, NEX Team Inc (San Jose, CA, USA) offers a small camera-based console (Nex Playground) that connects to a television via HDMI, allowing one to 4 players to interact with virtual games using body movements. Similarly, the MetFutAR Motion Gaming Console (MetaFuture Technologies Inc) connects to televisions via HDMI and uses built-in motion-sensing cameras to track body movements during gameplay. These systems overlay digital elements onto the real-world environment, effectively transforming living rooms into interactive play or activity spaces. Easy installation and relatively low cost are commonly promoted as key advantages of these technologies. These characteristics suggest that similar plug-and-play configurations may offer a practical and scalable platform for delivering AR-supported home-based rehabilitation.

AR systems may enhance the home exercise experience by providing interactive visual cues, gamified tasks, or virtual coaching, thereby increasing engagement and enjoyment. These systems can incorporate motion tracking and sensor-based feedback to provide immediate information on exercise performance, such as identifying errors in joint range of motion or movement quality, capabilities that paper exercise booklets cannot offer [17,33]. AR systems also create opportunities for remote or asynchronous monitoring by clinicians, enabling exercise feedback and progression without requiring frequent in-person visits. By supporting individualized exercise prescription and reducing reliance on in-person visits, AR-enabled home rehabilitation may help address geographic and transportation barriers that can limit access to post-TJR physiotherapy services [34].

Existing rehabilitation research on AR systems includes those targeting improved balance and muscle strengthening, falls prevention, upper limb functionality, and enhanced cognition [35]. Qualitative research has also begun to examine stakeholder perspectives on AR tools designed for home-based use. For example, Fields and colleagues explored the acceptability and appropriateness of an AR home assessment tool, highlighting the importance of user-centered design and stakeholder engagement when developing AR-supported home technologies for older adults [36]. A recent pilot trial of an interactive AR exercise system for knee and hip arthroplasty found that patients and therapists preferred the system over standard paper-based home exercise instructions [17]. Study participants highlighted the system’s potential to improve exercise accuracy and adherence to home exercise programs. While AR tools show considerable promise for enhancing TJR rehabilitation, limited engagement of key stakeholders in co-designing, implementing, and evaluating these technologies has hindered their translation into routine rehabilitation care [37,38].

The successful integration of any AR tool into routine rehabilitation care depends on careful alignment with end user needs and real-world clinical workflows [39]. Both older adults and their health care service providers (eg, physiotherapists) are key stakeholders whose perspectives could guide the design of an AR tool to support TJR rehabilitation. Engaging patients and health care service providers can illuminate desirable features, acceptable interfaces, and potential barriers, ensuring that the AR tool is developed to meet users’ needs and increases the potential for successful implementation in routine rehabilitation practice. This responds to a gap identified in the ARS literature: that heightened patient understanding of the features and functionality of an AR system is vital to creating beneficial, personalized rehabilitation tools [35].

Therefore, the objective of this study was to identify priorities for design features and practical or logistical considerations to inform the future design of a 3D AR system (3D ARS) to support home-based rehabilitation for older adults following TJR.

## Methods

### Overview

We began this research program by conducting a qualitative exploration of older adults’ perspectives, with particular focus on their pre- and postoperative experiences with TJR [40]. In-depth interviews and focus groups were conducted with 42 older adults with TJR and 4 unrelated family caregivers to explore their everyday use of technology, along with the potential role of AR tools to support home-based rehabilitation post-TJR [19]. Two key themes were identified: (1) dreaming up possibilities related to features, considerations, and logistics that could be incorporated into an ARS, such as providing customized feedback, including motivating messages while performing the exercises, and ensuring timely access to the ARS post-TJR surgery; and (2) being pragmatic about possible barriers to access and use of an ARS system, ranging from cost, confidence, and motivational challenges to concerns about accessing suitable home exercise equipment to maximize recovery while using the ARS [19].

Building on the qualitative findings, the themes informed the development of the survey in this study, which focused on prioritizing system features and outlining practical and logistical considerations to guide the design of a proposed 3D ARS to support home-based exercise programming for older adults after TJR.

As part of the survey development process, an expert-by-experience advisory panel (2 older adults with lived experience and 2 service providers) provided feedback on the survey items. The panel members were sent a draft of the survey and asked to review and complete it independently. After the draft surveys were completed and returned to the research office, a virtual meeting was convened to discuss item clarity, wording, readability, ease of access, and, importantly, overall relevance to older adults. Based on this feedback, revisions were made to refine the wording of survey items and improve clarity. The order of items was also revised based on the feedback to improve readability and flow. The updated survey was subsequently shared with panel members. No additional revisions were suggested at this stage.

Survey participants were recruited through flyers distributed by professional membership organizations (eg, the Canadian Physiotherapy Association, the Canadian Society for Exercise Physiology), organizations that offer support to people with arthritis (eg, the Arthritis Society of Canada), local libraries, seniors’ centers, and via emails to participants in our previous research who had consented to be re-contacted for future studies.

The inclusion criteria comprised individuals who self-identified as: (1) an older adult aged ≥65 years with a TJR within the last 6 months—to capture those with relevant post-TJR rehabilitation experience, or (2) a service provider (defined as a health care professional or community service provider with paid or volunteer experience working with older adults with TJR in formal care or community-based settings). All participants were further required to be residents of Canada.

Interested individuals scanned a QR code on the recruitment flyer to access the survey. After an online screening via Qualtrics, interested individuals were provided with detailed information about the study. All individuals were required to provide informed consent before completing the survey online. Each question enabled voluntary responses. Individuals who completed a survey could opt in to receive a $40 CAD (US $30) e-gift card. All survey data were collected using an institutional Qualtrics account (Silver Lake, Seattle, Washington, 2024). The survey took between 30‐45 minutes to complete.

### Data Collection

The survey consisted of 4 sections. Section 1 collected information on general sociodemographic and/or clinical characteristics. Sections 2 to 4 included items derived from our previous qualitative study [40] and were designed to gather information to guide the design and implementation of the 3D ARS.

In Section 2, participants were presented with a list of potential design features and asked to prioritize them for inclusion in the 3D ARS. Participants completed a forced rank-ordering task by dragging and dropping each feature into a ranked list from extremely important (rank=1) to least important (rank=14). Each feature was assigned a unique rank, and ties were not permitted. This approach produces relative, ordinal ranking data rather than independent Likert-type ratings.

Sections 3 and 4 consisted of structured questions with predefined response options using a “select all that apply” format, allowing participants to identify multiple relevant responses. Section 3 asked participants to indicate practical considerations and logistical preferences for the proposed AR system (eg, preferred feedback format, timing of motivational messages, and access pathways). Section 4 presented a list of potential barriers to AR system use and asked participants to select all options that applied to their experience or perspective.

Participants were also provided with a narrative description and a graphic depiction of the proposed 3D ARS, along with a glossary of key terms (eg, AR), to provide context and support informed responses.

### Data Analysis

Data were downloaded from Qualtrics and imported into SPSS version 29 (IBM SPSS Statistics) for analysis. Descriptive statistics, including frequencies and percentages, were used to summarize participant characteristics. Items using a “select all that apply” format were also analyzed descriptively using frequencies and percentages.

For survey items involving rankings of desired system features, differences between older adults with TJR and service providers were examined using Mann-Whitney *U* tests. This nonparametric test was selected because the ranking data were ordinal and the 2 groups were independent. Statistical significance was set at *P*<.05.

Since the survey allowed participants to skip individual questions, analyses were conducted using available-case analysis for each item. As a result, the number of responses varies slightly across analyses depending on the number of participants who completed each question.

### Ethical Considerations

Ethical approval of the online survey was obtained from the Queen’s University Health Sciences and Affiliated Teaching Hospitals Research Board (REH-858‐23). All participants provided informed e-consent before completing the survey. The survey was anonymous and no identifiable patient-related information was collected. Participants had the right to withdraw from the study by not completing the survey or by contacting the research team after submission to request that their data be withdrawn, without any adverse consequences. All study procedures were in accordance with the relevant guidelines/regulations, including data privacy and the Declaration of Helsinki. Participants who completed the survey and who opted in received a $40 CAD (US $30) e-gift card as a token of appreciation.

## Results

### Sample Characteristics

Table 1 summarizes the characteristics of the 145 participants who provided demographic information (out of 164 who activated the survey; 88.4% response rate). The sample consisted of 56 older adults with TJR and 89 service providers. Across the sample, most participants were male (68/145, 46.9%). Approximately one-third of the sample lived or worked in Ontario (47/145, 32.4%). Among older adults, left knee replacement was the most reported type of TJR (22/56, 39%). Older adults had a high level of education, with about one-third (22/56, 39%) holding a master’s degree. About one-third of the service providers were kinesiologists or exercise specialists (27/89, 30%). Most service providers were practicing in community-based settings (52/89, 58%).

**Table 1.** Participants’ characteristics (n=145)^a^.

Variable	Older adult (n=56)	Service providers (n=89)
Gender, n (%)		
Male	28 (50)	40 (45)
Female	28 (50)	18 (20)
Nonbinary	—^b^	11 (12)
Transgender man	—	12 (14)
Transgender woman	—	7 (8)
Gender fluid	—	1 (1)
Age (years), n (%)		
60‐65	10 (18)	—
66‐70	19 (34)	—
71‐75	14 (25)	—
76‐80	9 (16)	—
81‐85	3 (5)	—
86‐90	1 (2)	—
Province of residence, n (%)		
Alberta	3 (5)	10 (11)
British Columbia	11 (20)	9 (10)
Manitoba	2 (4)	20 (23)
New Brunswick	1 (2)	20 (23)
Newfoundland and Labrador	—	4 (5)
Nova Scotia	3 (5)	5 (6)
Ontario	36 (64)	11 (12)
Prince Edward Island	—	3 (3)
Quebec	—	6 (7)
Saskatchewan	—	1 (1)
Type of community, n (%)		
Rural area (population less than 1000)	2 (4)	5 (6)
Small center (population 1000‐29,000)	11 (20)	29 (33)
Medium center (population 30,000‐99,000)	9 (16)	45 (51)
Large urban center (population 100,000 or more)	34 (60)	4 (5)
Prefer not to disclose	—	3 (3)
Highest education, n (%)		
High school or general educational development	5 (9)	—
Technical or trade school	2 (4)	—
College	7 (13)	—
Bachelor’s degree	14 (25)	—
Master’s degree	22 (39)	—
Doctoral degree	6 (11)	—
Family income (US $)		
20,000‐49,999	13 (32)	—
50,000‐99,999	15 (27)	—
100,000‐149,999	8 (14)	—
150,000 or more	11 (20)	—
Prefer not to disclose	9 (16)	—
Type of joint replacement within the last 5 years, n (%)		
Left hip primary replacement surgery	7 (13)	—
Left hip revision surgery	2 (4)	—
Right hip primary replacement surgery	12 (21)	—
Right hip revision surgery	7 (13)	—
Left knee primary replacement	22 (39)	—
Left knee revision surgery	2 (4)	—
Right knee primary replacement surgery	19 (34)	—
Right knee revision surgery	1 (2)	—
Left ankle primary replacement surgery	4 (7)	—
Left ankle revision surgery	4 (7)	—
Right ankle primary replacement surgery	8 (14)	—
Right ankle revision surgery	2 (4)	—
Family caregiver during postoperative care, n (%)		
No one	1 (2)	—
Spouse	30 (54)	—
Friend	2 (4)	—
Offspring	10 (18)	—
Extended family members	10 (18)	—
Other	3 (5)	—
Service provider profession, n (%)		
Physiotherapist	—	10 (11)
Occupational therapist	—	21 (24)
Kinesiology or exercise specialist	—	27 (30)
Recreational therapist	—	8 (9)
Physician or physiatrist	—	1 (1)
Nurse	—	6 (7)
Social worker	—	6 (7)
Personal support worker	—	9 (10)
Prefer not to disclose	—	1 (1)
Types of technologies regularly used, n (%)		
Smartphone	51 (91)	75 (84)
SmartTV	35 (63)	82 (92)
Conventional TV	15 (27)	10 (11)
Smartwatch	21 (38)	17 (19)
Tablet	35 (63)	29 (33)
Wireless earphones	25 (45)	37 (42)
Laptop computer	35 (63)	31 (35)
Desktop computer	26 (46)	7 (8)
Other	4 (7)	—

a Percentages do not add up to 100% because participants could select all options that apply.

b Not applicable.

### Key Features

The Mann-Whitney *U* test revealed no significant differences between groups in rankings of “capability to embed individualized/tailored recommended exercise options in the 3D ARS” (*U*=2134.50, *Z*=−1.18; *P*=.24), “capability of the 3D ARS to deliver customized feedback on progress with recommended exercises” (*U*=2045, *Z*=−1.60; *P*=.11), or “capability to include background music during exercise” (*U*=2118.50, *Z*=−1.20; *P*=.23). However, older adults and service providers differed in their rankings of “capability of the 3D ARS to deliver positive encouraging messages while performing recommended exercises’” (*U*=1525, *Z*=−3.93; *P*<.001), “capability to activate the 3D ARS using voice activation prompts” (*U*=1145, *Z*=−5.39; *P*<.001), and “ability to pause and resume 3D ARS use at any time” (*U*=1843.50, *Z*=−2.51; *P*=.01)*.* Descriptive statistics for the overall rankings of desired features are presented in Table 2.

**Table 2.** Differences between older adults and service providers in ranking of desired features for the 3D augmented reality system.

Design features (rank ordered based on older adults’ and service providers’ responses)^a^ (n=142)	Older adults (n=55), n (%)	Median (IQR)	Service providers (n=87)	Median (IQR)	Mann-Whitney *U* test	Z statistic	*P* value
Individualized or tailored exercise options	40 (71)	1 (1-3)	80 (90)	2 (2-2)	2134.50	−1.18	.24
Positive encouragement delivered through the system while performing recommended exercises	30 (54)	2 (1-3)	77 (87)	2 (1-3)	1525	−3.93	<.001
Customized feedback on progress with recommended exercises	29 (52)	2 (1-3)	13 (15)	3 (0-3)	2045	−1.60	.11
Ability to pause and continue system use at any time	28 (50)	2 (1-3)	19 (21)	3 (3-3)	1843.50	−2.51	.01
System availability 24/7	21 (38)	3 (1-5)	10 (11)	4 (4-4)	1971	−1.87	.06
Embedded technical support to problem-solve issues or difficulties with system use	19 (34)	3 (2-4)	13 (15)	5 (4-5)	1346.50	−4.59	<.001
Background music while performing recommended exercises	12 (21)	4 (3-6)	16 (18)	4 (3-4)	2118.50	−1.20	.23
Interactive gaming features	9 (16)	7 (5-8)	1 (1)	8 (8-8)	1109	−6.84	<.001
System connection to other personal digital devices	8 (14)	5 (4-7)	3 (3)	6 (6-6)	1778.50	−3.02	.003
Availability of various language options	7 (13)	6 (4-6)	7 (8)	6 (6-6)	1655.50	−4.04	<.001
System connection and interactivity with other sources of support	6 (11)	6 (4-7)	1 (1)	7 (7-7)	1610	−3.82	<.001
System activation using voice activation prompts	5 (9)	5 (4-5)	48 (54)	2 (1-5)	1145	−5.39	<.001
Choice and control over on-screen images presented	3 (5)	4 (3-5)	26 (29)	4 (2-5)	1734.50	−2.85	.004
Choice and control over the virtual environment	3 (5)	5 (4-6)	34 (38)	5 (3-7)	1194.50	−5.26	<.001

a Total sample of 145 with 3 values missing.

### Practical Logistics for the 3D ARS

**Figure 1.**
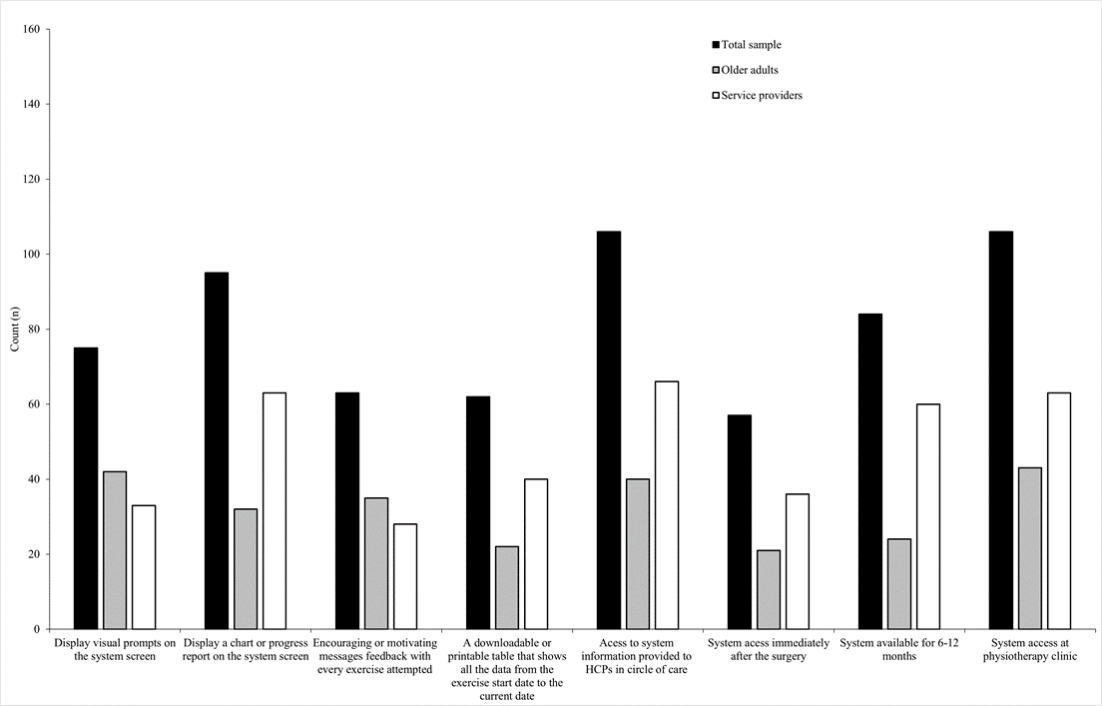
Practical considerations and logistical details for using the 3D augmented reality system. HCP: health care professional.

Figure 1 summarizes practical considerations and logistical details for the 3D ARS. When asked how customized feedback should be delivered, most older adults with TJR (42/56, 75%) preferred visual prompts displayed on the 3D ARS screen to help guide and adjust joint range of motion. Conversely, most service providers (63/89, 71%) preferred a chart report of joint range-of-motion exercises displayed on the 3D ARS screen.

Regarding how encouraging or motivational messages should be delivered, most older adults with TJR (35/56, 63%) and about one-third of service providers (28/89, 31%) preferred motivational messages delivered after every exercise attempt. For tracking and documentation, about one-third of older adults (22/56, 39%) and nearly half of the service providers (40/89, 45%) favored a downloadable or printable table that shows all the data from the exercise start date to the current date.

Regarding where and when end users should obtain access, and who should have data access, most older adults with TJR (43/56, 77%) and service providers (63/89, 71%) recommended that users obtain access to the 3D ARS through a physiotherapy clinic. In terms of timing, over one-third of older adults (21/56, 38%) and nearly half of the service providers (42/89, 47%) indicated that access should begin immediately after surgery or within 4 weeks post-surgery. Nearly half of older adults (24/56, 43%) and more than two-thirds of service providers (60/89, 67%) recommended maintaining access for 6‐12 months. Additionally, most older adults (40/56, 71%) and service providers (66/89, 74%) recommended that health and social care providers within the user’s circle of care should have access to information generated through the 3D ARS.

### Potential Barriers to Access and Usage of the 3D ARS

Table 3 summarizes the potential barriers to access and use of the 3D ARS. As shown in the table, over half of the older adults (31/56, 55%) and service providers (48/89, 54%) reported that cost would be a potential barrier. Nearly half of the older adults (25/56, 45%) and service providers (42/89, 47%) indicated that lack of access to suitable exercise equipment to perform recommended exercises at home would also be a top barrier. Among older adults, additional barriers included a lack of confidence in the 3D ARS setup and use (31/56, 55%) and a lack of motivation to use the 3D ARS (26/56, 46%). Service providers did not identify additional top barriers.

**Table 3.** Barriers to access and use of 3D augmented reality system (3D ARS; n=145).

Barriers	Older adult (n=56), n (%)	Service providers (n=89), n (%)
Cost of accessing the 3D ARS	31 (55)	48 (54)
Lack of confidence to set up and use 3D ARS	31 (55)	28 (31)
Lack of motivation to use 3D ARS	26 (46)	20 (23)
Lack of access to appropriate equipment to support exercising at home	25 (45)	42 (47)
Lack of adequate space in residence	21 (38)	30 (34)
Overdependence on 3D ARS for recovery	20 (36)	25 (28)
Inability to complete exercises unsupervised at home	20 (36)	6 (7)
Privacy and safety concerns	15 (27)	33 (37)

## Discussion

### Principal Findings

This study examined priorities for a 3D ARS to support home-based physical rehabilitation for older adults recovering from TJR. Overall, our findings indicate that older adults were open to the potential use of a 3D ARS to support postoperative rehabilitation in the home. Participants emphasized the importance of individualized exercise programming, timely and meaningful feedback during exercises, and user-friendly system features that support ease of interaction. While older adults and service providers shared several priorities for system design, differences emerged in preferences for certain features, including the format and timing of feedback, motivational messaging, and options that support user autonomy, such as control over on-screen images and virtual environments. Together, these findings highlight key user-informed considerations that can guide the development of AR-based rehabilitation technologies for older adults following TJR.

### User Priorities and Personalization in AR-Based Rehabilitation

Older adults and service providers shared several priorities for the design of the 3D ARS, including individualized exercise programs, customized feedback on exercise progress, continuous system availability, and optional features such as background music. At the same time, differences emerged in preferences for certain features, particularly those related to motivational messaging and user autonomy. Participants emphasized the importance of the system’s ability to deliver positive encouragement during exercise and to provide options that allow users greater control over on-screen images, virtual environments, and language settings. These findings reflect the heterogeneous needs and recovery experiences of older adults following TJR [41] and underscore the importance of tailoring rehabilitation programming to individuals’ functional capacities. Personalization has been widely identified as a core design principle in geriatric rehabilitation technologies, supporting engagement and sustained participation in home-based exercise programs [42].

### Real-Time Feedback and the Role of Automated Support in Home-Based Rehabilitation

A key finding was the difference in preferred feedback formats during exercises. Service providers favored simple charts and progress reports, whereas older adults preferred immediate, on-screen visual prompts. This distinction is consistent with previous research showing that therapists often prefer concise, actionable summaries rather than navigating extensive patient metrics generated by digital rehabilitation systems [43]. Prioritizing older adults’ perspectives as end users highlights the importance of integrating real-time feedback into AR-based rehabilitation technologies. Although previous studies have demonstrated positive responses to synchronous feedback from remote therapists during exercise sessions [17], the shortage of therapists, logistical challenges of scheduling virtual supervision, and associated health care costs may limit the feasibility of such approaches in routine rehabilitation care [44]. While synchronous supervision may enhance safety for certain clinical populations requiring hands-on support [45], AR-based systems that provide automated feedback may offer a more scalable solution for supporting home-based rehabilitation. Another important finding related to the timing of feedback was that participants preferred motivational messages to be delivered after each exercise attempt. This finding aligns with existing evidence suggesting that frequent positive feedback can reinforce desired behaviors and improve exercise adherence [46]. This finding emphasizes the need to incorporate multimodal feedback mechanisms that accommodate both patient needs and provider oversight requirements in the design of the 3D ARS.

### Usability and User Experience Considerations for 3D ARS for Older Adults

Beyond feedback mechanisms, participants emphasized the importance of system usability and ease of interaction when engaging with AR-based rehabilitation technologies. In particular, features such as voice activation and the ability to pause and resume exercises were identified as important for supporting interaction with the 3D ARS. Significant differences between older adults and service providers also emerged for these usability-related features, suggesting that flexibility in how users interact with and control the system may be an important consideration in the design of home-based rehabilitation technologies. Usability is a critical determinant of engagement with digital health technologies, particularly among older adults [47]. Technologies that are difficult to navigate can create confusion and frustration, which may reduce sustained engagement and limit the effectiveness of digital rehabilitation interventions [48,49]. Designing AR-based systems that prioritize intuitive interaction and flexible system control may therefore support greater engagement and usability in home-based postoperative rehabilitation.

### Privacy-Preserving Motion Tracking and Data Sharing Considerations in AR-Based Rehabilitation

In addition to usability considerations, participants and system designers must also consider privacy and data management issues associated with camera-based rehabilitation technologies. We propose the use of artificial intelligence–based pose estimation as a promising approach for balancing the need for meaningful movement analysis with concerns related to privacy and data management in home-based rehabilitation. By converting video input into a simplified 3D skeletal representation of the body, the system will capture key movement information while removing identifiable visual features such as facial characteristics, clothing, and background details. This approach may help address privacy concerns that often accompany camera-based monitoring technologies, which can be a barrier to adoption in home environments. In addition, the system focuses on the individual closest to the camera, thereby minimizing the unintended capture of other individuals in the home.

Beyond privacy considerations, the skeletal representation substantially reduces the amount of data generated during exercise sessions compared with full video streams. This reduction in data volume facilitates efficient storage and transmission of exercise information and supports the feasibility of local data storage directly on the console. Such efficiency may be particularly important for in-home rehabilitation contexts where internet connectivity or bandwidth may be limited.

Importantly, this approach also enables flexible models of information sharing with health care providers. At the most basic level, individuals can retain full control over what information is shared by verbally communicating their progress or challenges during follow-up consultations. Alternatively, the system could generate automated summaries of exercise performance, including indicators such as repetitions completed, trends in performance, or potential anomalies. These summaries could be shared manually or electronically through secure communication channels. When more detailed monitoring is required, anonymized skeletal data could also be transmitted to clinicians, enabling more precise review of exercise performance while maintaining a high level of privacy protection.

Taken together, these features suggest that artificial intelligence–based pose estimation may offer a viable pathway for integrating motion tracking into AR-based home exercise systems in a manner that supports both patient privacy and clinical oversight [50]. Such approaches may help address key implementation challenges associated with remote rehabilitation technologies and support more scalable models of home-based care [51,52].

### Clinical Integration and Access to AR-Based Rehabilitation

Our findings raise important considerations regarding health equity in the implementation of technology-based rehabilitation. Physiotherapy clinics may vary in their capacity to integrate and support technology-enabled interventions over extended periods, and not all patients may have equal access to such resources due to geographic, financial, or systemic barriers [53]. Addressing these challenges during system development and implementation will be critical to ensuring that the benefits of the 3D ARS are scalable and equitably distributed.

Within this context, participants recommended that access to the 3D ARS be facilitated through physiotherapy clinics, initiated early in the postoperative period, and maintained for at least 6 months. This finding aligns with previous work highlighting physiotherapists’ central role and expertise in exercise prescription for older adults with TJR [54]. Situating access within clinical settings may also ensure that the use of the 3D ARS is guided by professional oversight, reducing the risk of misuse while positioning the system as a complement to conventional rehabilitation care.

### Cost, Digital Equity, and Access to Rehabilitation Technologies

Other issues identified include the potential cost of the 3D ARS and a lack of access to suitable exercise equipment to complete the recommended exercises at home. Overall, participants’ perspectives on costs highlight issues of digital equity and the importance of efforts to address the existing divide in access to digital infrastructure, which gives some individuals advantages over others [55]. To address these concerns, the proposed 3D ARS will leverage a relatively simple hardware configuration similar to existing motion-based gaming consoles, in which a camera-based console connects directly to a television or monitor. Such an approach could facilitate easy integration into home environments while minimizing installation complexity. By using commonly available household electronics, such as a TV or monitor, this configuration may also help reduce system costs and improve the feasibility of implementing AR-based rehabilitation technologies in the home.

### Supporting Independent Use of the 3D ARS Through Accessible System Design

Finally, the lack of confidence in the 3D ARS setup identified by older adults highlights the importance of incorporating robust training supports and designing intuitive, user-friendly setup procedures [56]. Embedding step-by-step guidance, interactive tutorials, and a clear user interface design within the system itself could increase user confidence while reducing cognitive load and reliance on external assistance. Such design choices not only empower users to engage more independently but may also help minimize system development and implementation costs by reducing the need for dedicated technical support staff to resolve setup issues.

Overall, these findings highlight several key considerations for the development of AR-based rehabilitation technologies for older adults following TJR. Participants emphasized the importance of systems that combine individualized exercise programming, accessible and intuitive user interfaces, and timely feedback mechanisms that support motivation and adherence. At the same time, our results underscore the need to balance technological capabilities with practical implementation considerations, including privacy protection, clinical oversight, and equitable access to digital rehabilitation resources. By incorporating these user-informed priorities into the design of the 3D ARS, future systems may better align with the needs of both patients and rehabilitation professionals, supporting scalable models of home-based rehabilitation that complement conventional care.

### Limitations

Several limitations should be considered when interpreting the findings of this study. First, participants were asked to evaluate proposed features of a 3D ARS rather than interacting with a fully functional system. As a result, the preferences expressed may not fully reflect experiences with actual system use during postoperative TJR rehabilitation. Future studies should examine user experiences with prototype systems to better understand usability, engagement, and clinical effectiveness in real-world contexts.

Second, the sample included a relatively highly educated group of older adults, which may limit the generalizability of findings to individuals with lower levels of education or digital literacy. Because comfort with technology can influence perceptions of digital health tools, the perspectives captured in this study may not fully represent the broader population of older adults undergoing TJR.

Third, the cross-sectional design captures participant perspectives at a single point in time and does not account for how preferences may evolve during different stages of postoperative recovery or with increased familiarity with digital rehabilitation technologies. Longitudinal research may provide additional insight into how user needs and expectations change throughout the rehabilitation process.

Finally, although participants were recruited from multiple regions, a substantial proportion of the sample was located in Ontario. Health system structures, access to rehabilitation services, and availability of digital infrastructure may vary across regions, which could influence the transferability of these findings to other settings.

### Conclusions

This study identified key design priorities for a 3D ARS to support home-based physical rehabilitation for older adults recovering from TJR. These findings provide an important foundation for the development of AR-based rehabilitation technologies that respond to the needs of end users and rehabilitation professionals. Future work will focus on user-centered system development, prototype testing, and usability evaluation to support the implementation of a practical and scalable AR-based rehabilitation solution.

## Abbreviations

3D ARS: 3D augmented reality system
AR: augmented reality
TJR: total joint replacement
VR: virtual reality
